# Emergency Medicine Challenges in Ecuador

**DOI:** 10.5811/westjem.2020.8.47694

**Published:** 2020-10-28

**Authors:** Andrés M. Patiño, Santiago Cantillo-Campos, Alexis S. Kearney, Sean M. Kivlehan, Augusto Maldonado

**Affiliations:** * Emory University School of Medicine, Department of Emergency Medicine, Atlanta, Georgia; † Duke University, Department of Emergency Medicine, Durham, North Carolina; ‡ Brown University, Department of Emergency Medicine, Providence, Rhode Island; § Brigham and Women’s Hospital, Department of Emergency Medicine, Boston, Massachusetts; ¶ Universidad San Francisco de Quito, Hospital General Docente Calderon, Department of Emergency Medicine, Quito, Ecuador

## Abstract

**Introduction:**

Emergency medicine (EM) was recognized as a specialty in Ecuador in 1993. Currently, there are two four-year EM residency programs and an estimated 300 residency-trained emergency physicians countrywide. This study describes the current challenges in EM in Ecuador.

**Methods:**

We conducted 25 semi-structured, in-person interviews with residency-trained emergency physicians, general practitioners, public health specialists, prehospital personnel, and physicians from other specialties. The interviewer asked about challenges in the areas of emergency care, working conditions of emergency physicians, EM residency education, EM leadership, and prehospital care. We analyzed data for challenges and registered the number of interviewees who mentioned each challenge.

**Results:**

Interviewees worked in the three largest cities in the country: Quito (60%); Guayaquil (20%); and Cuenca (20%). Interviewees included 16 (64%) residency-trained emergency physicians; six (24%) residency-trained physicians from other specialties working in or closely associated with the emergency department (ED); one (4%) general practitioner working in the ED; one (4%) specialist in disasters; and one (4%) paramedic. Shortage of medical supplies, need for better medico-legal protection, lack of EM residencies outside of Quito, and desire for more bedside teaching were the challenges mentioned with the highest frequency (each 44%). The next most frequently mentioned challenges (each 38%) were the need for better access to ultrasound equipment and the low presence of EM outside the capital city. Other challenges mentioned included the low demand for emergency physicians in private institutions, the lack of differential pay for night and weekends, need for more training in administration and leadership, need for a more effective EM national society, and lack of resources and experience in EM research.

**Conclusion:**

Emergency medicine has a three-decade history in Ecuador, reaching important milestones such as the establishment of two EM residencies and a national EM society. Challenges remain in medical care, working conditions, residency education, leadership, and prehospital care. Stronger collaboration and advocacy among emergency physicians can help strengthen the specialty and improve emergency care.

## INTRODUCTION

Ecuador is a small, upper-middle income country located in northwestern South America. It has a population of approximately 17 million.^1^ Quito, the capital, and Guayaquil are the most populous cities with approximately 2.7 million inhabitants each.^2^ The country’s gross domestic product per capita in United States dollars is $11,500; approximately 21% of the population lives beneath the poverty line.^1^,^3^ Emergency medicine (EM) was recognized as a specialty in Ecuador in 1993 and has reached significant milestones since. This qualitative, descriptive statistical analysis presents the history and current state of EM in Ecuador and identifies current challenges with the goal of informing future EM development work in the country.

## BACKGROUND

### Healthcare System

Ecuador provides universal healthcare through a mixed public-private health system.^4^ Overall, 30% of Ecuadorians have Social Security Insurance (employees working in the formal economy), 58% seek medical care in public facilities (individuals who have low income or work informal jobs), and 12% have private health insurance.^4^ Healthcare resources vary greatly among medical facilities in Ecuador, from small outpatient facilities in rural areas with minimal resources to large tertiary-care hospitals in the larger cities. Private facilities are usually better resourced than public ones.

General practitioners still provide most emergency care in the country, either independently at lower acuity facilities, or under the supervision of emergency physicians (EP) or other specialists in tertiary-care centers. Most residency-trained EPs work in Quito, with fewer than 50 estimated to work in other cities, including Guayaquil, Cuenca, Ambato, Manta, and Portoviejo. By law, all emergency departments (ED) must care for patients with life-threatening emergencies, regardless of their insurance status or ability to pay but are not obligated to provide any additional care once the patient has been stabilized. The majority of EDs employ the Manchester triage system.^5^

### Medical Education

Medical school education begins immediately after high school, and programs are offered by both public and private universities. All programs are six years in length, plus one year of social service in underserved areas after graduation. General practitioners may then work independently or under the supervision of specialists or apply to residency programs.*

**Population Health Research Capsule**

What do we already know about this issue?

*Emergency medicine (EM) has been recognized as a specialty in Ecuador for the last three decades, and there are two EM residency programs in the country*.

What was the research question?

*This study sought to identify current challenges in the specialty and the state of emergency care in Ecuador*.

What was the major finding of the study?

*Important challenges remain in delivering medical care, as well as in EM working conditions, residency education, leadership, and prehospital care*.

How does this improve population health?

*This qualitative study can serve as the starting point for future EM research and development of the specialty in Ecuador*.

### Emergency Medicine History in Ecuador

In 1986 the Ecuadorian Society of Emergency Medicine and Disasters (SEMED) was formed by general practitioners interested in emergency care. Shortly thereafter in 1989, author AM’s interest in EM was piqued during a rotation in the ED while still early in his pulmonary medicine residency. Inspired by the US EM curricula, AM modified his residency curriculum and graduated in 1993 as the first residency-trained EM specialist in the country. He subsequently helped establish the first residency program in EM and Disasters at Universidad Central and later founded the residency program at Universidad Católica. A third program, affiliated with Universidad San Francisco de Quito only lasted a few years due to differences between the university and the main clinical site.

At the time of this study there were an estimated 300 EPs in Ecuador.^6^ Most completed EM residency training, while a minority were grandfathered into the specialty after working in EDs for at least five consecutive years. This mechanism was also used in other specialties and was terminated by the government in 2000. At the time of this study, SEMED had 20 members, all of whom were EM specialists.

### Emergency Medicine Residencies

There are two EM residency programs in Ecuador: Universidad Central and Universidad Católica, a private university (Table 1). Both programs are four years in length and are located in Quito. Admission processes for each institution require a school-specific written exam, a certificate of intermediate English proficiency, and an in-person interview. Both residency programs charge tuition, but few residents pay the full amount. Instead, tuition and stipends for residents are sponsored by the government or hospitals where they rotate. In addition to clinical rotations (Table 2), both residencies have extensive didactic activities including several hours of lectures every week with mandatory attendance. Both programs use medical simulation and require residents to document medical procedures in a logbook. Ultrasound training curricula is the same for both residency programs.

Following residency, graduates who received government scholarships must repay their financial aid by working two years for every year of financial aid received in government-designated hospitals, often in other cities or rural locations. This method of repayment is referred to as *devengar* (“to earn”) and applies to graduates of all specialties. There are no board exams for EM or any other specialty in Ecuador. Due to the relatively small number of residency-trained EPs in Ecuador, many graduates go on to be the first EPs hired by their institutions, and often find themselves in leadership roles.

### Prehospital Care

The national emergency medical services (EMS) system is called Servicio Integrado de Seguridad ECU 911 (ECU-911 for short). The system is activated by dialing 911. ECU-911 is a mixed public-private system. Most ambulances are staffed with paramedics, while very few employ physicians.^7^ Paramedic training is a four-year undergraduate degree. General practitioners provide medical control in call centers. No EPs were involved in medical control at the time of this study. Although ECU-911 is a nationwide initiative, prehospital capabilities vary widely among different regions. In 2018 Quito’s ECU-911 response included 16 ambulances. Nationwide protocols published by the Public Health Ministry exist to guide EMS and prehospital emergency care.^7^

### Pediatric Emergency Medicine

General practitioners and pediatricians provide most of the emergent health care to the pediatric population as there are no fellowship programs for pediatric EM in the country. Residents from both EM residencies rotate through Hospital Baca Ortiz, a large referral pediatric hospital in Quito, and are supervised by pediatricians and pediatric critical care specialists.

## METHODS

In April 2018, 25 semi-structured, in-person interviews were conducted with EM specialists, general practitioners, prehospital personnel, public health specialists, and physicians from other specialties in 10 EDs across the three largest cities in Ecuador: Quito, Guayaquil, and Cuenca. All interviews were performed by author AP and consisted of an initial list of 13 general questions and 23 questions related to EM training (see Online Supplement A). The questions were asked in an open-ended manner, with follow-up questions as needed. Non-EM specialists were included in the sample given their current role in the provision of emergency care in Ecuador and with the aim of including diverse points of view. Interview questions were developed based on prior experience with EM development research^8^,^9^ and focused on the current challenges in the areas of emergency care, EM working conditions, EM education, EM leadership, and prehospital care.

Questions related to EM working conditions, EM leadership, and EM training were directed to EM specialists only. Only EM specialists were asked questions about residency training. This included individuals who were grandfathered into EM as well as those who were residency trained. The initial interviewees were identified by author AM based on personal and professional contacts. We used snowball chain-referral sampling to identify subsequent participants. The recruitment email included a study fact sheet, and informed consent was implied by voluntary completion of the interview. Challenges were identified and the number of interviewees who mentioned each challenge was recorded. Interviews and data analysis were conducted in Spanish. Challenges mentioned by three or more respondents were translated for inclusion in the manuscript. The authors performing the interviews and data analysis are fluent in both Spanish and English. Ethical approval was obtained from the Partners Healthcare Institutional Review Board (IRB), in Boston, MA, and the Hospital General Docente Calderón IRB, in Quito, Ecuador.

## RESULTS

### Interviewee Characteristics

All 25 subjects approached participated in an interview (Table 3). Fifteen (60%) participants worked in Quito, and the majority (64%) were EPs. With respect to current employment, 60% of participants worked exclusively in the clinical setting, 12% in healthcare administration only, and 28% in both. Approximately one-third of the interviewees were directors of their EDs.

### Challenges

Interviewees cited many challenges with respect to the provision of emergency care and EM as a specialty. Table 4 lists the challenges mentioned by at least three interviewees. Themes were divided into five categories: emergency care; EM working conditions; EM education; EM leadership; and prehospital care.

## DISCUSSION

EM has reached important milestones in Ecuador over the last three decades. However, important challenges remain. The most frequently mentioned challenge in emergency care was the shortage of medical supplies. Some interviewees attributed this to a lack of expertise of managers, mismanagement of funds, and funding variability with political cycles. ED crowding and long wait times were also mentioned. ED crowding is not unique to Ecuador.^10^ One potential strength of the Ecuadorian system is that the overwhelming majority of its healthcare facilities are public or part of the nation’s Institute of Social Security, which could allow for easier implementation of reforms at a national level as compared to more decentralized systems. Interviewees also identified the need for better application of protocols for the management of time-sensitive pathologies, both in the hospital and the prehospital settings. Some EDs lack protocols. Others have them on paper but do not apply them, resulting in delays and inefficiencies.

With respect to working conditions, interviewees reported medical lawsuits are increasing in Ecuador and felt a lack of medico-legal protection was negatively affecting the specialty. EP compensation was also mentioned by interviewees as an issue. EPs work a disproportionate number of night and weekend shifts, caring for critically ill patients in a stressful environment, and balancing the needs of multiple patients at the same time.^11^,^12^ However, all medical specialists working in government or Social Security hospitals are paid the same, regardless of specialty or shift distribution. Interviewees mentioned that few EPs are employed by private hospitals. In these institutions, the payment model favors multiple specialist consultations, encouraging general practitioners or EPs to consult other specialties for conditions within the EP’s scope of practice. This results in inefficiencies, increased cost, and increased length of stay. Lack of access to bedside ultrasound equipment was also listed as a challenge under working conditions, as it is an important clinical tool for the specialty. It could have also been listed under the emergency care category as lack of bedside ultrasound access can affect the overall care of patients.

The two most frequently mentioned challenges in EM education were the absence of residency programs outside of Quito and an increased desire for bedside teaching and supervision during training. A new EM residency is planned in Cuenca, which could help expand the national visibility of EM. Bedside teaching is limited by EM specialists being only available part of the day in some training hospitals. Increased EM specialist coverage, increased senior resident teaching, additional time in the simulation center, or telemedicine initiatives could be potential alternatives to increase supervision.

The most salient challenge in EM leadership was the lack of EM presence outside of Quito. EM specialists are gradually starting to work in other cities. Another EM leadership challenge mentioned was the need for a stronger EM national society. At the time of data collection, SEMED had a small membership and limited involvement and advocacy in EM issues. Active recruitment of EM specialists and EM residents, programming that appeals to EPs, and allowing full voting rights and involvement in leadership for new members could increase transparency and participation. A strong EM society can advocate for its members and provide guidance to hospitals, government officials, and other healthcare entities about issues affecting emergency care and EPs.^13^ Another challenge in EM leadership was the lack of EM research. While all EM residents are required to complete a research project, the quality of the projects is variable. Few EP-led projects are published in international medical journals. Potential reasons for low research output mentioned by interviewees included heavy clinical and teaching loads of academic faculty, limited funding opportunities, and lack of role models and experience in EM research locally.

The challenges most frequently mentioned in prehospital care were the lack of strong protocols for time-sensitive conditions, difficulties and delays in transfer to tertiary hospitals, and lack of EP involvement. Prehospital care in Ecuador would benefit from further involvement of EM specialists to improve coordination between the prehospital and ED settings and to implement systemwide protocols for time-sensitive conditions. Despite the many challenges identified by this study, EM continues to grow in Ecuador. Addressing the issues identified here could help expedite the growth of the specialty and improve emergency care for Ecuadorians.

### Future Directions

After completion of this study, the authors, SEMED, and the American College of Emergency Physicians (ACEP) co-sponsored the First Forum About the Future of Emergency Medicine in Ecuador in September 2019, in Quito. The conference drew over 80 participants from around the country, including many EM residents. The goals of the forum were as follows: 1) share the results of this research project; 2) bring together Ecuadorian EM specialists to further discuss current challenges in EM and identify possible solutions; and 3) to expose Ecuadorian EM specialists to international EM leaders to promote the transnational flow of ideas. Speakers included Ecuadorian EPs and international EM experts. SEMED presented a reform plan that included the following: active recruitment of new members with full voting rights for EM specialists; free membership for EM residents; and formal collaborations with international EM organizations. SEMED also proposed the formation of task forces to address multiple issues, including education and government relations. At the time of submission of this publication, SEMED’s membership had increased to more than 80.

## LIMITATIONS

An important limitation of this study is the small sample size, which was due to time constraints. However, as seen in Table 4, many themes were mentioned by multiple participants. The small sample size limited the ability to conduct subgroup analyses by city or by specialty. Similarly, not all respondents were able to answer questions for all topics (eg, only EM specialists answered questions about EM training, EM working conditions, and EM leadership). Additionally, only challenges that were mentioned by three or more respondents were included to increase reliability, which may have resulted in the exclusion of important minority opinions. Our findings are likely most limited for the prehospital care themes given that our sample was largely composed of EM specialists who currently have little involvement in the prehospital system in Ecuador.

The referral chain-sampling method may have inserted bias. A survey of all EPs in Ecuador could have allowed for more robust statistical analysis; however, there is no central repository of contact information for all EM specialists in Ecuador. Furthermore, little information exists about EM as a specialty or its challenges in Ecuador, which made it difficult to develop a detailed survey. Instead, a semi-structured interview approach was pursued to permit follow-up questions that could result in a deeper understanding of themes and context.

Despite these limitations, this study provides important details about the history and current challenges of EM in Ecuador and has already initiated conversations and reform among local EPs. The issues identified may not apply to every hospital in Ecuador, and local context must be considered. Ultimately, Ecuadorian EPs are best positioned to address these challenges.

## CONCLUSION

Emergency medicine has a three-decade history in Ecuador, reaching important milestones such as the establishment of two EM residencies and a national EM society. Challenges remain in medical care, working conditions, residency education, leadership, and prehospital care. Increased involvement of emergency physicians in administrative and leadership roles and stronger advocacy on both a local and national level will help strengthen the specialty and improve emergency care.

## Supplementary Information



## Figures and Tables

**Table 1:** Emergency medicine residency program characteristics in Ecuador.

Program	Universidad Central	Universidad Católica
Year founded	1994	2004
City	Quito	Quito
Institution type	Public university	Private university
Duration	4 years	4 years
Tuition per year	USD $5,000*	USD $7,000*
Stipend	USD $1,600*	USD $900 – $1,600*
Class size	15–18	20–25
Total residents	35	65
EM faculty	50	30
Application requirements	Written exam English exam Interview	Written exam English exam Interview
Fellowships offered	None	None
Special Features	Oldest program in the country	ACLS and ATLS certifications included for residents

* Tuition and stipend usually paid by scholarship

*EM*, emergency medicine; *USD*, United States dollar; *ACLS*, Advanced Cardiovascular Life Support; *ATLS*, Advanced Trauma Life Support.

**Table 2:** Ecuadorian emergency medicine residency curricula: number of months spent in each clinical rotation.

Program	Universidad Central months in rotation	Universidad Católica months in rotation
Emergency Medicine	23 (12 in critical EM)	16
Pediatrics	3	2
ICU	6	12
Other rotations	EM Observation 4	Anesthesia 2
	Internal Med-Cardiology 4	Cardiology 2
	Neurologic Emergencies 4	Gastroenterology 2
	Pulmonology Emergencies 4	Internal Med 4
	Ultrasound*	Neurology 2
		Prehospital 2
		Ultrasound*
		International or Provincial Rotation 2–4

* Ultrasound training consists of 30 hours of lecture and 100 hours of supervised practice for both programs over four years.

*EM*, emergency medicine; *ICU*, intensive care unit.

**Table 3:** Characteristics of subjects who were interviewed about the state of emergency care in Ecuador.

	Number	%
City (n = 25)		
Quito	15	60%
Guayaquil	5	20%
Cuenca	5	20%
Specialty (n = 25)		
Emergency medicine	16	64%
Surgery	2	8%
Pediatrics	2	8%
General practitioner	1	4%
Disasters	1	4%
Internal medicine	1	4%
Critical care	1	4%
Paramedic	1	4%
Work setting (n = 25)		
Only clinical	15	60%
Only administrative	3	12%
Both	7	28%
Sector (n = 25)		
Public	23	91%
Private	2	9%
ED director (n = 25)	9	36%

*ED*, emergency department.

**Table 4:** Emergency medicine challenges in Ecuador.

	Number of interviewees who mentioned challenge	% of interviewees who mentioned challenge
Emergency care (n = 25)		
Shortages of medical supplies	11	44%
Longer wait times	7	28%
Crowding and boarding	7	28%
Need for stronger application of institutional protocols for time-sensitive conditions (eg. stroke, MI, trauma)	6	24%
Emergency medicine working conditions (n = 16, emergency medicine specialists only)		
Need for better medico-legal protection	7	44%
Need for increased access to bedside ultrasound	6	38%
Low demand for emergency physicians in private institutions	5	31%
Lack of differential pay for night and weekend shifts	5	31%
Emergency medicine education (n =16, emergency medicine specialists only)		
Absence of postgraduate programs outside the capital city	7	44%
Desire for more bedside teaching and supervision during residency	7	44%
Interest in more training in administration and leadership	5	31%
Government scholarship repayment (Devengar)	3	19%
Lack of emergency medicine subspecialty fellowships (eg, ultrasound)	3	19%
Emergency medicine leadership (n = 16, emergency medicine specialists only)		
Low presence and recognition of EM outside of capital city	6	38%
Need for a more effective EM national society	5	31%
Few resources and lack of experience in EM research	5	31%
Prehospital care (n = 25)		
Lack of strong prehospital protocols for time-sensitive conditions	4	16%
Difficulties and delays in referrals to tertiary care medical centers	4	16%
Lack of involvement of emergency physicians in the prehospital system	3	12%

*MI*, myocardial infarction; *EM*, emergency medicine.

## Footnotes

* In Ecuador, physicians pursuing a medical specialty degree are referred to as *posgradistas* or “postgraduates,” instead of “residents,” the term more commonly used for these trainees in other countries. In contrast, in Ecuador the term “resident” is used for general practitioners working under the supervision of a specialist, without actively pursuing a specialty degree. Given that this article is aimed at an international audience the terms “resident” and “residency” will be used in their more common international usage, as opposed to their Ecuadorian meaning.
