# Supplementary material for: Emergency Medicine Challenges in Ecuador
Source: West J Emerg Med. 2020 Oct 28;21(6):284–90. doi: 10.5811/westjem.2020.8.47694 (PMC7673876; doi:10.5811/westjem.2020.8.47694)
Supplement: Supplementary file 1 [file wjem-21-284-s001.docx]

| **ONLINE SUPPLEMENT A**  **Questionnaire for Semi-Structured Interviews**  **General Questions**   1. What is the history of emergency care and emergency medicine in your organization and in Ecuador? 2. What are some of the strengths of the way emergency care is currently provided in your organization and in Ecuador? 3. What are some of the needs in emergency care in your organization and in Ecuador? 4. What are some of the opportunities for improvement of emergency care in your organization and in Ecuador? 5. What are some of the strengths and weaknesses of in medical and emergency medicine education in Ecuador? 6. What are some of the threats to the development of emergency medicine in Ecuador? 7. What qualifications are required to be an emergency care provider? 8. Which kinds of physicians staff the emergency department? 9. In what settings do emergency medicine physicians practice? (riding ambulance, ICU, ED, etc.) 10. What are some of the organizations providing leadership in emergency care? 11. How does emergency care differ between urban and rural areas? 12. How is pre-hospital care provided in Ecuador? 13. What types of emergency medicine research projects are taking place in Ecuador?   **Additional Questions for Residency Program Personnel**   1. Year founded: 2. University affiliation: 3. City: 4. Length: 5. Tuition (amount): 6. Resident salary (if any, amount): 7. Application process:    1. Nacional test score range____________    2. Medical Written test (yes/no)____________    3. English test (written, spoken, both, neither)___________    4. Interviews (how many, with whom)    5. Other requisites? 8. Number of applicants every year: 9. Residents accepted every year: 10. Percent graduation rate (aka how many drop out) 11. Current number of residents in each year: 1_____, 2_______, 3________, 4________ 12. Clinical Sites: List all clinical sites EM sites (not off service rotations)  \| Hospital \|  \|  \|  \|  \|  \| \| --- \| --- \| --- \| --- \| --- \| --- \| \| # ED beds \|  \|  \|  \|  \|  \| \| # Total beds \|  \|  \|  \|  \|  \| \| # ED visits per year \|  \|  \|  \|  \|  \| \| Admission rate \|  \|  \|  \|  \|  \| \| Other info \|  \|  \|  \|  \|  \|  1. EM Fellowships offered (e.g. Ultrasound, critical care, EMS, etc): 2. Number of months dedicated to rotations    1. Adult EM:    2. Pediatric EM:    3. ICU:    4. Step down    5. Obstetrics    6. Ward medicine    7. Ward surgery    8. Ultrasound:    9. Emergency medical services:    10. Toxicology:    11. Antesthesia:    12. Trauma surgery:    13. Other rotations (and # of months spent in them): 3. Other curriculum characteristics:    1. Number of didactic hours per week:    2. Format of didactic hours (e.g. 3 hours of lecture, 1 hour of simulation ):    3. Number of simulation hours per month    4. Research Requirement/thesis/ academic project: yes___, no______       1. Type of product expected at the end (poster, article, etc)    5. Does the residency require ACLS, ATLS, PALS? Yes____, No___       1. Who covers the cost?    6. Procedure log? Yes____ No ____    7. Resident evaluation:       1. Written exams? Yes____ No ____. Format? ____ How often?______       2. Practical exam? Yes____ No ____. Format? _____ How often? ____       3. Feedback to residents? Yes____ No ____How often?____ 4. Graduation test or board certification test? Yes____ No ____. Which ones? ____    1. Passing rate? 5. Number of EM-trained Faculty: full time_________, part time__________ 6. EM faculty to resident ratio: 7. Protected academic time for faculty? 8. Is EM its own department in the institution? If not, under which department? 9. Areas of research: 10. Challenges faced by faculty? 11. Challenges faced by residents? |
| --- | --- | --- | --- | --- | --- | --- | --- | --- | --- | --- | --- | --- | --- | --- | --- | --- | --- | --- | --- | --- | --- | --- | --- | --- | --- | --- | --- | --- | --- | --- | --- | --- | --- | --- | --- | --- |
